# TLR2, TLR4 and the MYD88 Signaling Pathway Are Crucial for Neutrophil Migration in Acute Kidney Injury Induced by Sepsis

**DOI:** 10.1371/journal.pone.0037584

**Published:** 2012-05-24

**Authors:** Angela Castoldi, Tárcio Teodoro Braga, Matheus Correa-Costa, Cristhiane Fávero Aguiar, Ênio José Bassi, Reinaldo Correa-Silva, Rosa Maria Elias, Fábia Salvador, Pedro Manoel Moraes-Vieira, Marcos Antônio Cenedeze, Marlene Antônia Reis, Meire Ioshie Hiyane, Álvaro Pacheco-Silva, Giselle Martins Gonçalves, Niels Olsen Saraiva Câmara

**Affiliations:** 1 Disciplina de Nefrologia, Departamento de Medicina, Universidade Federal de São Paulo, São Paulo, Brazil; 2 Laboratório de Imunobiologia de Transplantes, Departamento de Imunologia, Universidade de São Paulo, São Paulo, Brazil; 3 IIEP, Hospital Israelita Albert Einstein, São Paulo, Brazil; 4 Patologia, Universidade Federal do Triângulo Mineiro, Uberaba, Brazil; University of Colorado Denver, United States of America

## Abstract

The aim of this study was to investigate the role of TLR2, TLR4 and MyD88 in sepsis-induced AKI. C57BL/6 TLR2^−/−^, TLR4^−/−^ and MyD88^−/−^ male mice were subjected to sepsis by cecal ligation and puncture (CLP). Twenty four hours later, kidney tissue and blood samples were collected for analysis. The TLR2^−/−^, TLR4^−/−^ and MyD88^−/−^ mice that were subjected to CLP had preserved renal morphology, and fewer areas of hypoxia and apoptosis compared with the wild-type C57BL/6 mice (WT). MyD88^−/−^ mice were completely protected compared with the WT mice. We also observed reduced expression of proinflammatory cytokines in the kidneys of the knockout mice compared with those of the WT mice and subsequent inhibition of increased vascular permeability in the kidneys of the knockout mice. The WT mice had increased GR1^+low^ cells migration compared with the knockout mice and decreased in GR1^+high^ cells migration into the peritoneal cavity. The TLR2^−/−^, TLR4^−/−^, and MyD88^−/−^ mice had lower neutrophil infiltration in the kidneys. Depletion of neutrophils in the WT mice led to protection of renal function and less inflammation in the kidneys of these mice. Innate immunity participates in polymicrobial sepsis-induced AKI, mainly through the MyD88 pathway, by leading to an increased migration of neutrophils to the kidney, increased production of proinflammatory cytokines, vascular permeability, hypoxia and apoptosis of tubular cells.

## Introduction

Severe sepsis is the major cause of acute kidney injury (AKI) (2–4) . Despite all efforts to better comprehend this pathology, little progress has been achieved. This might be due to the fact that most research groups have focused more on proving that AKI is mainly caused by changes in kidney hemodynamics, while other groups have shown the importance of non-hemodynamic factors in the establishment of this disease, such as immunological factors [1], [2]. The kidney damage after sepsis is likely a result of these two important contributions, starting with the recognition of bacterial products by Toll-like receptors (TLRs), which recognize pathogens, such as PAMPs (*pathogen associated molecular patterns*) and are responsible for the inflammatory cascade in sepsis.

Signal transduction through TLRs is partially mediated by a TLR adapter protein MyD88 (*Myeloid Differentiation Factor 88*). MyD88 can bind to the TIR domain and recruit signaling proteins, leading to the activation of transcription factors, such as NF-κB (*Nuclear Factor- kappa B*) and AP-1 (*Activator Protein 1*), and the expression of genes related to the inflammatory response, such as tubular necrosis factor TNF-α, interleukin- 6 and IL-1β.

Several studies have shown the importance of TLRs in the development of sepsis, but few studies have correlated the activation of TLRs with the development of AKI. During sepsis, TLRs are activated by bacteria and endogenous ligands such as HMGB-1 and HSP70, which are released during cellular stress, and interact with the immune system in the extracellular environment [3], [4]. These alarmins exacerbate the kidney inflammatory process during sepsis, which results in greater damage and necrosis.

Thus, we hypothesized that mice deficient in TLR2 and TLR4 and the adaptor protein MyD88 respond less effectively to bacteria, leading to a milder inflammatory process and therefore weaker protection of renal function compared with wild-type mice.

## Materials and Methods

### Mice

We used male C57BL/6 mice aged between 6 and 8 weeks, weighing 20 g to 28 g, which were genetically deficient (^−/−^) for TLR2, TLR4 and MyD88. The animals were provided by the animal facility of the Center for Development of Experimental Models for Medicine and Biology CEDEME-UNIFESP. The animals were housed at the Nephrology Division, UNIFESP, in cages containing a maximum of five animals, with the artificial light/dark 12-hour cycle at a constant temperature of 22°C and supplemented with water and food *ad libitum*.

### Cecal ligation puncture (CLP) model

For the induction of sepsis, the animal's cecum was punctured twice with a 23-gauge needle, followed by light compression to ensure the removal of intestinal contents. The animals were killed 24 hours after sepsis induction.

### Assessment of renal function

Serum creatinine was measured by Jaffé's modified method. Serum urea was measured using a Labtest Kit (Labtest, Minas Gerais, Brazil).

### RNA extraction and Real-Time PCR

Total RNA was isolated using the Trizol Reagent (Invitrogen, Carlsbad, CA). First-strand cDNAs were synthesized using the MML-V reverse transcriptase kit (Promega, Madison, Wisconsin, USA). Reverse transcriptase polymerase chain reaction (PCR) was performed using TaqMan probes. mRNA expression was normalized to HPRT. The values were expressed relative to a reference sample (the calibrator) and sham-operated samples. The Ct (threshold cycle) for the target gene and for the internal control were determined for each sample. Samples were run in triplicate. The relative expression of each mRNA was calculated by 2^−ΔΔCT^. All experimental sample values are expressed as the n-fold difference relative to the calibrator. The primers used are described in Data S1 and S2.

### Histology and quantification of ATN

The quantification of acute tubular necrosis (ATN) was performed with H&E (Hematoxylin and Eosin) staining, and analysis was performed using the computer program Image Pro Plus for Windows (USA) coupled to an optical microscope Olympus BX40F-3 (Olympus Optical Company, Japan) for capturing and digitizing the images of the fields evaluated. For the quantitative analysis, we used the computer program Image Lab (São Paulo, Brazil). Results were expressed as the percentage of area affected (selected) in relation to the total area of the field measured (0.073 cm^2^). We chose 20 fields randomly in an increase of 20 times per slide. We studied five animals in each group. The analysis was performed in such a way that the observer had no access to material identification.

### Flow cytometry

The kidneys were harvested, opened, macerated and sieved (70–100 µm) with cold RPMI. The cells were resuspended in a solution of DNAse/Collagenase (1 mg/2 mg per mL) and later separated by Percoll gradient. The peritoneal lavage was collected after CLP and centrifuged. Samples (∼2 million cells) were then collected, centrifuged and resuspended in 20 mL of FACS buffer. We then labeled the cells with anti-GR1 APC and anti-F4/80 PERCP monoclonal antibodies for the analysis of surface molecules and anti-TNF-α PE for the analysis of the intracellular molecule (BD Biosciences). The samples were analyzed using a FACSCanto device using the FACSDIVA software (BD Biosciences) and then analyzed with the software FlowJo (Tree Star, San Carlo, CA). Background fluorescence was determined using unlabeled cells, and compensation was performed using cells stained with APC, PERCP and PE. We analyzed 1,000,000 events.

### Assessment of renal microvascular protein leakage using Evans blue dye

The microvascular leakage was assessed with Evans blue dye as previously described [5], [6]. The amount of Evans blue dye was analyzed by measuring the absorbance at 620 nm. Results were calculated from a standard curve of Evans blue dye and expressed as micrograms of Evans blue dye per mg of kidney (wet weight).

### Immunohistochemistry (IHC)

Pimonidazole (Chemicon International, Inc., CA, USA) was administrated intraperitoneally at a dose of 60 mg/kg 1 hour before sacrifice and detected by the Hypoxyprobe-1 Pab2627 (1∶500) primary antibody as described previously [5]. Immunohistochemistry with cleaved caspase-3 antibody (diluted 1∶1000 (Asp175), Cell Signaling Technology, Beverly, MA, USA) was also performed as previously described [7]. Immunohistochemistry for NF-κB p65 was also performed (#8242, diluted 1∶100, Cell Signaling Technology, Beverly, MA, USA). The presence of primonidazole-HCL , cleaved caspase-3 and NF-κB p65 in renal tissue was quantitated as a percentage in the cortex and medulla using a computer program for image analysis (KS300, Zeiss system). The average area of each sample was calculated for each kidney.

### Assessment of apoptosis

To detect apoptotic cells, the *In situ* Cell Death Detection Kit TMR red (Roche Diagnostics GmbH, Mannheim, Germany) was used (TUNEL technology).

### Detection of Myeloperoxidase (MPO) in renal tissue

MPO in renal tissue was estimated as previously described by Hillegass et al. [8]. The reading was performed in a spectrophotometer at a wavelength of 460 nM.

### Western blotting analysis

Primary mouse IKKα antibody (SC-166231, Santa Cruz Biotechnology, Inc) was used following manufacturer-recommended dilutions, followed by a peroxidase-conjugated anti-mouse IgG antibody (Jackson ImmunoResearch Laboratories, WestGrove, USA). Mouse primary anti–β-tubulin or anti-β-actin antibody (Sigma, St. Louis, USA) was also used to confirm and estimate the loading and the transfer. We used the software GeneSnap (Syngene, USA) and Gene Tools (Syngene, USA) to analyze the bands.

### Neutrophil depletion

Purified GR1 antibody RB6-8C5 (DNAX Research Institute, Palo Alto, CA, USA) was obtained from a hybridoma culture supernatant. To deplete the mice of neutrophils, a single dose of 0.25 mg was administered intraperitoneally 24 hours before sepsis. Treatment with this dose of antibody induced severe neutropenia for up to 5 days.

### Bacteria count in the peritoneal cavity

Quantitative bacterial culture was performed for peritoneal colony-forming units (CFU) of control mice and 24 hours after sepsis induced by CLP. The CFU were determined after serial dilution, and culture medium agar was inoculated with 50 microliters of 1×10^6^ CFU and incubated in an oven at 37°C for 18 h.

### CBA (Cytometric Bead Array)

Cytometric Bead Array (CBA) Mouse Th1/Th2/Th17 Cytokine Kit (BD Biosciences) was performed to quantify IL-6, TNF-α and IL-17 in the peritoneal fluid as described by manufacturer.

### ELISA

To analyze the secretion of IL-1β in the peritoneal cavity after sepsis, we used ELISA assay (R&D Systems, Minneapolis, MN, USA).

### Statistical analysis

The data are presented in graphs showing average and standard deviation (SD) or median and lower and upper ranges (histomorphometric analysis). T tests, the Mann-Whitney test and ANOVA on ranks tests were used to compare the data. The PCR results are presented as a ratio of the calibrator gene HPRT and presented in arbitrary units (AU). Differences were considered statistically significant with p less than 0.05. To study survival, the animals were monitored two times daily for 8 days (192 hours) after CLP. The long-rank test was used for analysis of the survival curve. All statistical analyses were performed with the aid of GraphPad PRISM®.

## Results

### MyD88 knockout improves survival after sepsis-induced AKI

Initially, we observed that there was an up-regulation of TLR2, TLR4 and MyD88 in the WT mice that were subjected to sepsis. We also observed that in the absence of TLR2, there is an over expression of TLR4. Similarly, in the absence of TLR4, there was an over expression of TLR2 (**Figure S1**).

**Figure 1:**
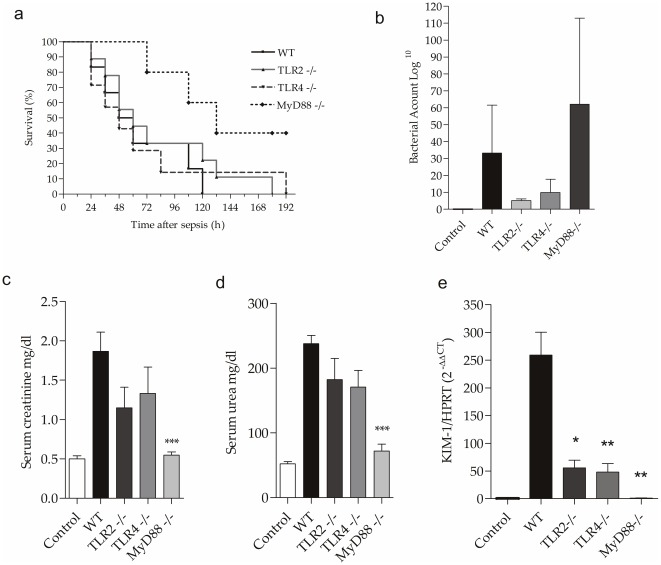
Effect of the absence of TLR2, TLR4 and MyD88 in the survival and in the development of acute Kidney Injury of animals subjected to CLP. (a) Survival of WT, TLR2^−/−^ , TLR4^−/−^ and MyD88^−/−^ mice subjected to sepsis by CLP. Mice were evaluated twice each day until the time 192 h after surgery. Results are representative experiment with 10 animals/group (WT vs MyD88^−/−^ p<0.05). (b) Bacterial count in the peritoneal cavity 24 hours after CLP . (c and d) Renal function of WT, TLR2^−/−^ , TLR4^−/−^ and MyD88^−/−^ mice 24 hours after CLP, assessed by levels of serum creatinine and blood urea. (e) Gene expression of KIM-1 in kidney of control and WT, TLR2^−/−^ , TLR4 ^−/−^ and MyD88^−/−^ mice 24 hours after CLP. The mRNA was normalized to HPRT expression and compared to normal group. Data shown as mean ± standard deviation (SD), *** p<0. 0001 vs WT; ** p<0.01 vs WT; *p<0.05 vs WT.

To determine whether the absence of TLR2, TLR4 and MyD88 affects the mortality in AKI induced by CLP, we evaluated the survival of all mice for 192 hours after the induction of sepsis. We observed that the MyD88^−/−^ mice had higher survival rates compared with other groups (p<0.05) (**Figure 1a**), but the bacterial count in the peritoneal cavity was higher in the MyD88^−/−^mice (**Figure 1b**).

**Figure 2:**
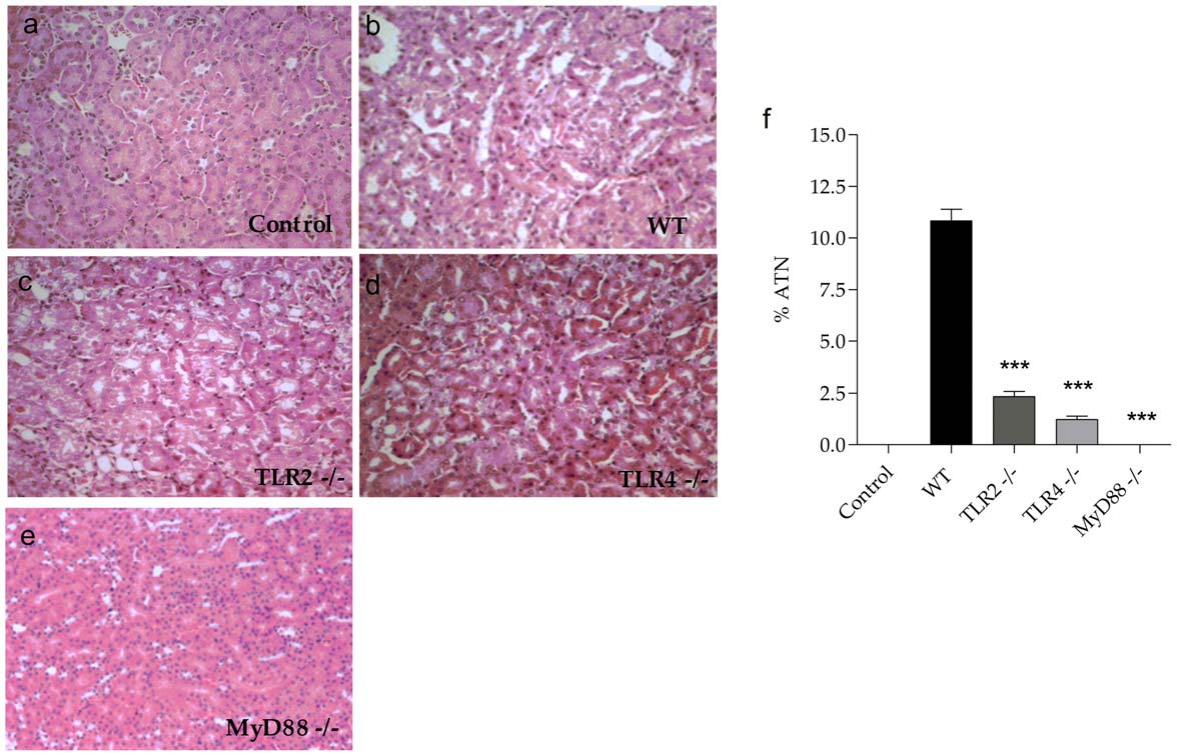
Acute tubular necrosis in TLR2, TLR4 and MyD88 deficient mice subjected to CLP. Histology and quantification of ATN of control(a) and WT(b), TLR2^−/−^ (c), TLR4 ^−/−^ (d) and MyD88^−/−^ mice (e) 24 hours after CLP. Results of a representative experiment with 5 animals per group. Data shown as mean ± standard deviation (SD), *** p<0.0001 vs WT.

Next, we observed that the MyD88^−/−^ mice were completely protected from renal dysfunction caused by sepsis, while the TLR2^−/−^ and TLR4^−/−^ animals only seemed to improve but did not reach statistical significance (**Figure 1c and 1d**). However, we observed a significant increase in KIM-1 (*kidney injury molecule-1*) gene expression in the WT mice and a decrease in the TLR2^−/−^ and TLR4^−/−^ mice and an even greater decrease in the MyD88^−/−^ mice (**Figure 1e**). Corroborating these data, the renal histology showed that the WT mice developed ATN with loss of brush borders and vacuolar degeneration 24 hours after CLP, while the TLR2^−/−^ and TLR4^−/−^ mice presented with less serious injury, demonstrating significant protection. Further, the MyD88^−/−^ mice showed no such histological changes (**Figure 2**), showing a strong protective effect of the lack of MyD88 on AKI.

### Sepsis induces expression of endogenous ligands in the kidney

**Figure 3:**
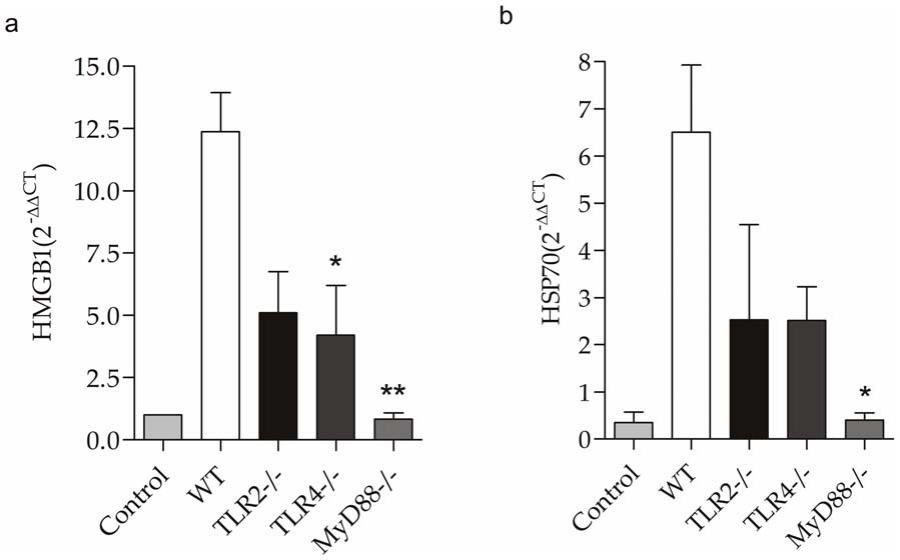
Endogenous ligands expression in kidney in animals subjected to CLP. mRNA expression of HMGB1 (a), HSP70 (b) in the kidney of WT, TLR2^−/−^, TLR4^−/−^ and MyD88^−/−^ mice 24 hours after CLP. The mRNA was normalized to HPRT expression and compared to normal group. Results of a representative experiment with 5 animals per group. Data presented as mean ± standard deviation (SD), * p<0.05 vs WT; ** p<0.01 vs WT.

TLR activation in the kidney during sepsis can be elicited by endogenous ligands called alarmins, such as HMGB1 and HSP70 [9]. We studied the mRNA levels of HMGB1 (**Figure 3a**) and HSP70 (**Figure 3b**) in the kidney of the control, WT, TLR2^−/−^, TLR4^−/−^ and MyD88^−/−^ mice 24 hours after CLP. We observed that the WT, TLR2^−/−^, and TLR4^−/−^ mice had increased expression of these two alarmins in the kidney after CLP. MyD88^−/−^ mice showed a decrease in endogenous ligand expression after CLP.

### TLR2, TLR4 and MyD88 deficiency is associated with lower renal expression of pro-inflammatory cytokines

**Figure 4:**
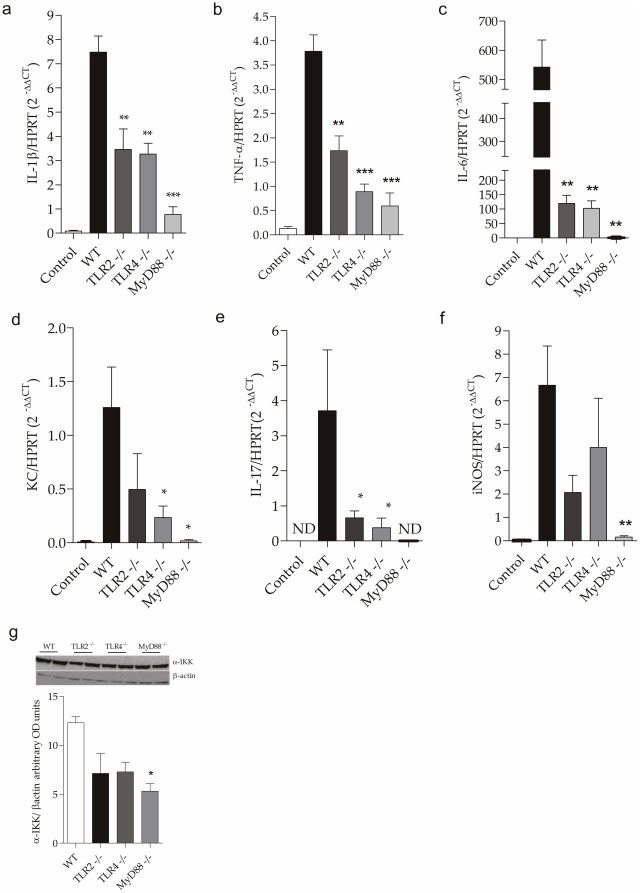
Expression of pro-inflammatory cytokines after CLP. mRNA expression of IL-1β (a), TNF-α (b), IL-6 (c), KC (d), IL-17 (e) and iNOS (f) in the kidney 24 hours after CLP. The mRNA was normalized to HPRT expression and compared to normal group. Results of a representative experiment with 5 animals per group. (g) Analysis of expression of α-IKK in kidney of WT, TLR2^−/−^ , TLR4^−/−^ and MyD88^−/−^ mice 24 hours after CLP. Results representative of a experiment with two animals/group Data shown as mean ± standard deviation (SD), * p<0.05, ** p<0.01 and *** p<0.001 vs WT.

TLR2 and TLR4 activation via MyD88 leads to the nuclear translocation of NF-κB and AP-1, resulting in the expression of genes related to the inflammatory response. TNF-α is a major pro-inflammatory molecule activated in experimental models of sepsis and is considered one of the main mediators of AKI [10]. We observed lower mRNA levels of IL1-β, IL-6 and TNF-α in TLR2^−/−^, TLR4^−/−^ and MyD88^−/−^ mice compared with WT (**Figure 4a, b and c**) 24 hours after CLP. Furthermore, we observed an increased expression of IL-17 and KC in the WT mice and reduced expression in the TLR2^−/−^ and TLR4^−/−^ mice, while in the MyD88^−/−^ mice, the expression was almost absent, suggesting that the absence of MyD88 causes reduced migration of neutrophils into the kidney after CLP (**Fig. 4 d and e**).

**Figure 5:**
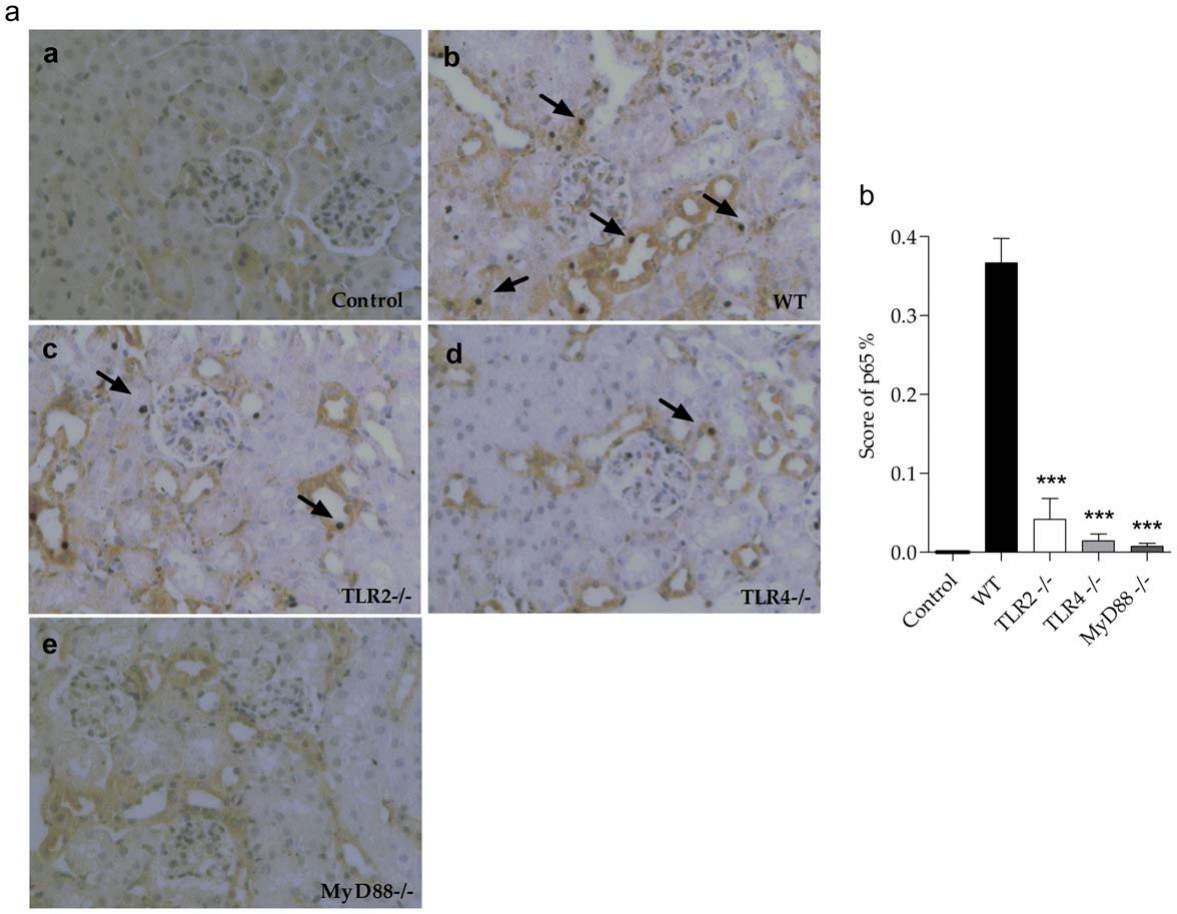
NF-κB activity in the kidney of TLR2, TLR4 and MyD88 deficient mice subjected to CLP. (**a**) Histological analysis of NF-κB p65 by immunohistochemistry of control (a), WT (b), TLR2 ^−/−^ (c), TLR4 ^−/−^ (d) and MyD88 ^−/−^ (e) mice respectively, 24 hours after CLP . Results of a representative experiment with five animals per group. (**b**) Score of NF-κB p65 in kidney represented in figure A. Data shown as mean ± standard deviation (SD), *** p<0.01 vs WT.

We also studied the gene expression of iNOS in kidney of these mice, since iNOS is considered an important factor that contributes to the pathogenesis of septic shock. We observed a significant decrease of iNOS in the knockout mice, especially in the MyD88^−/−^ mice (**Figure 4 f**). We also observed that the knockout mice subjected to CLP had lower activation of NF-κB compared with the WT mice (p<0.05) (**Figure 4g**). The significant decrease in inflammation in knockout mice seems to be a consequence of decreased NF-κB activity in the kidney. We observed by immunohistochemistry that the knockout mice were significantly protected from the translocation of NF-κB p65 into the nucleus compared to WT, thus culminating in decreased transcription of inflammatory molecules (**Figure 5a and b**).

### The absence of TLR2, TLR4 and MyD88 inhibits the increase in renal vascular permeability after CLP

**Figure 6:**
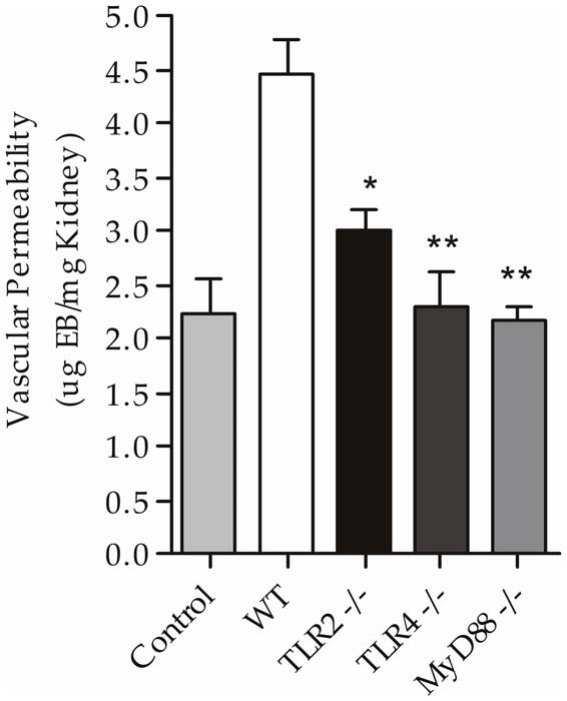
Renal vascular permeability in TLR2, TLR4 and MyD88 deficient mice subjected to CLP. Analysis of vascular permeability in the kidney of the control, WT, TLR2^−/−^, TLR4^−/−^ and MyD88^−/−^ mice 24 hours after CLP. Results are representative of an experiment with five animals per group. Data shown as mean ± standard deviation (SD), ** p<0.01 vs WT and * p<0.05 vs. WT.

We observed that sepsis induced an increase in vascular permeability in the kidneys of the WT mice (4.20±0.33 µg/mg); however, the TLR2^−/−^ (2.99±0.18 µg/mg), TLR4^−/−^ (2.31±0.31 µg/mg, p<0.01) and MyD88^−/−^ mice (2.37±0.22 µg/mg, p<0.01) had lower vascular permeability indexes compared with the WT septic mice, underscoring the renal protection already observed (**Figure 6**).

### Effects of sepsis on renal tubular hypoxia

**Figure 7:**
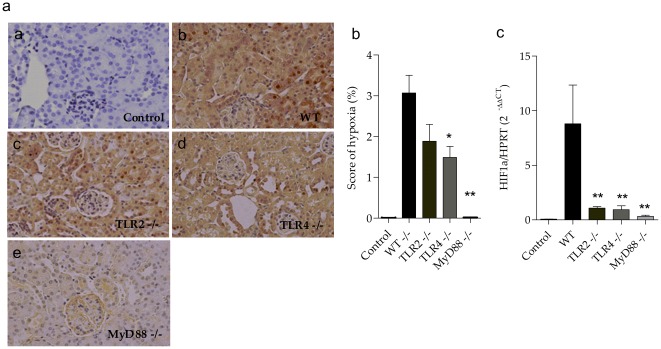
Renal hypoxia in TLR2, TLR4 and MyD88 deficient mice subjected to CLP. (a) Histological analysis of hypoxia by immunohistochemistry (IHC) of control (a), WT (b), TLR2^−/−^ (c) , TLR4^−/−^ (d) and MyD88^−/−^ mice (e) respectively, 24 hours after CLP. Results of a representative experiment with 5 animals per group. (b) Score of hypoxia level represented by iminohistochemistry in figure A. (c) Gene expression of HIF-1α in the kidney 24 hours after CLP. The mRNA was normalized to HPRT expression and compared to normal group. Results of a representative experiment with 5 animals per group. Data shown as mean ± standard deviation (SD), * p<0.05 vs WT and ** p<0.01 vs WT.

We analyzed whether the absence of TLRs and consequent decreased production of cytokines could lead to organ protection through lower tubular hypoxia. The presence of hypoxia was observed in cortical tubules but not in the medullar areas of the kidney. After CLP, we observed less areas of hypoxia in the TLR2^−/−^, TLR4^−/−^ and MyD88^−/−^ kidneys when compared to the control WT kidneys (**Figure 7a and b**). We also evaluated the presence of renal hypoxia by HIF1-α mRNA expression analysis and observed increased expression of this gene induced by hypoxia in the WT mice kidneys (**Figure 7c**).

### Effects of sepsis on apoptosis in the kidney

**Figure 8:**
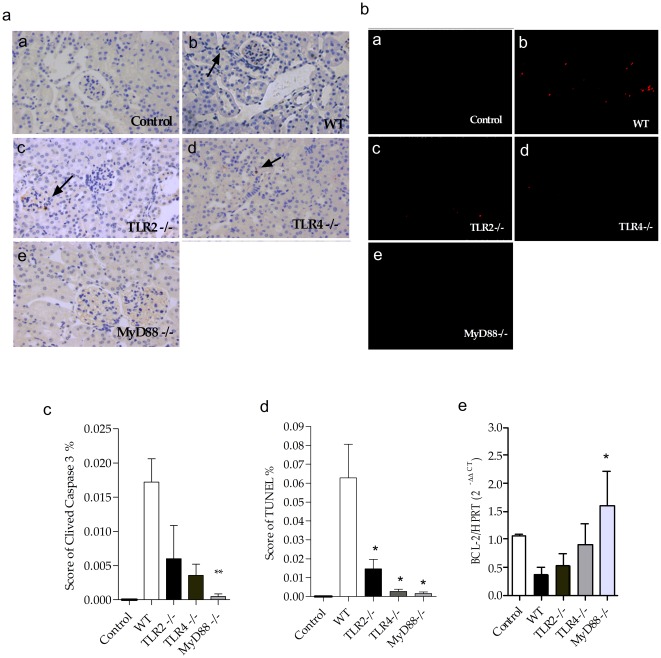
Apoptosis in the kidney of TLR2, TLR4 and MyD88 deficient mice subjected to CLP. (**a**) Histological analysis of apoptosis by immunohistochemistry of cleaved caspase 3 of control (a), WT (b), TLR2 ^−/−^ (c), TLR4 ^−/−^ (d) and MyD88 ^−/−^ (e) mice respectively, 24 hours after CLP . Results of a representative experiment with 5 animals per group. (**b**) Histological analysis of apoptosis by immunofluorescence to TUNEL of control (a), WT (b), TLR2 ^−/−^ (c), TLR4 ^−/−^ (d) and MyD88 ^−/−^ mice (e) respectively, 24 hours after sepsis. (**c**) Score of cleaved caspase-3 in kidney represented in figure A. (**d**) Score of TUNEL in kidney represented in figure B. (**e**) Gene expression of BCL-2 in the kidney 24 hours after CLP. The mRNA was normalized to HPRT expression and compared to normal group. Results of a representative experiment with 5 animals/group. Data shown as mean ± standard deviation (SD), * p<0.05 vs WT and ** p<0.01 vs WT.

Recent studies have shown that apoptosis may also be involved in the pathogenesis of sepsis [11]. We observed less cleaved caspase 3 in the tubular cells after CLP in the MyD88^−/−^ mice compared with those cells from the other mice (**Figures 8a and c**). The TUNEL assay showed a similar result (**Figures 8b and d**), but this method appeared to be more sensitive and showed that the WT mice underwent more apoptosis than the knockout mice. Interestingly, we observed that the MyD88^−/−^ mice had higher levels of the anti-apoptotic factor Bcl-2 than the WT mice (**Figure 8e**).

### Effects of TLR2, TLR4 and MyD88 knockout on secretion of TNF-α, IL1-β, IL-6 and IL-17 in the peritoneal cavity after CLP

**Figure 9:**
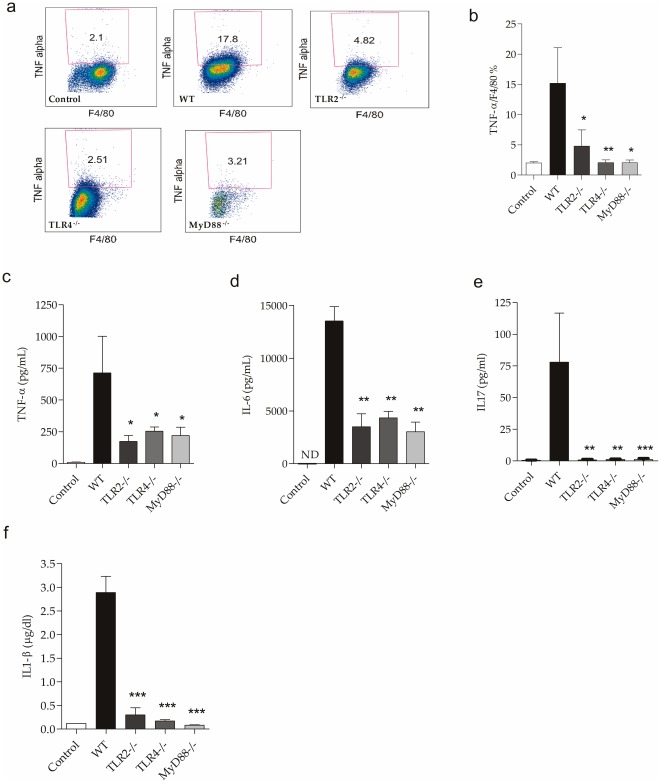
Secretion of TNF-α, IL-1β, IL-6 and IL-17 in the peritoneal cavity after CLP. (a) Flow cytometry of the peritoneal cavity 24 hours after CLP in control, WT, TLR2^−/−^, TLR4^−/−^ and MyD88^−/−^ mice. (b) Frequency of TNF-α/F4/80+ cells population in control, WT, TLR2^−/−^, TLR4^−/−^ and MyD88^−/−^ mice 24 hours after CLP.(c) Analysis of TNF-α, (d) IL-6, (e) IL-17 by CBA in control, WT, TLR2^−/−^, TLR4^−/−^ and MyD88^−/−^. (f) Analysis of IL-1β by ELISA in control, WT, TLR2^−/−^, TLR4^−/−^ and MyD88^−/−^ . Data shown as mean ± standard deviation (SD). * p<0.05 vs. WT, ** p<0.01 vs. WT and ***p<0.001 vs. WT.

We observed a significant increase in the production of TNF-α, IL-1β, IL-6 and IL-17 from the peritoneal cavity in the WT mice and a significant decrease in the knockout mice (**Figure 9a, b, c, d, e and f**). These findings corroborate the gene expression of these molecules in the kidneys of the septic mice.

### Effect of TLR2, TLR4 and MyD88 deficiency on migration of neutrophils to the peritoneal cavity after CLP

**Figure 10:**
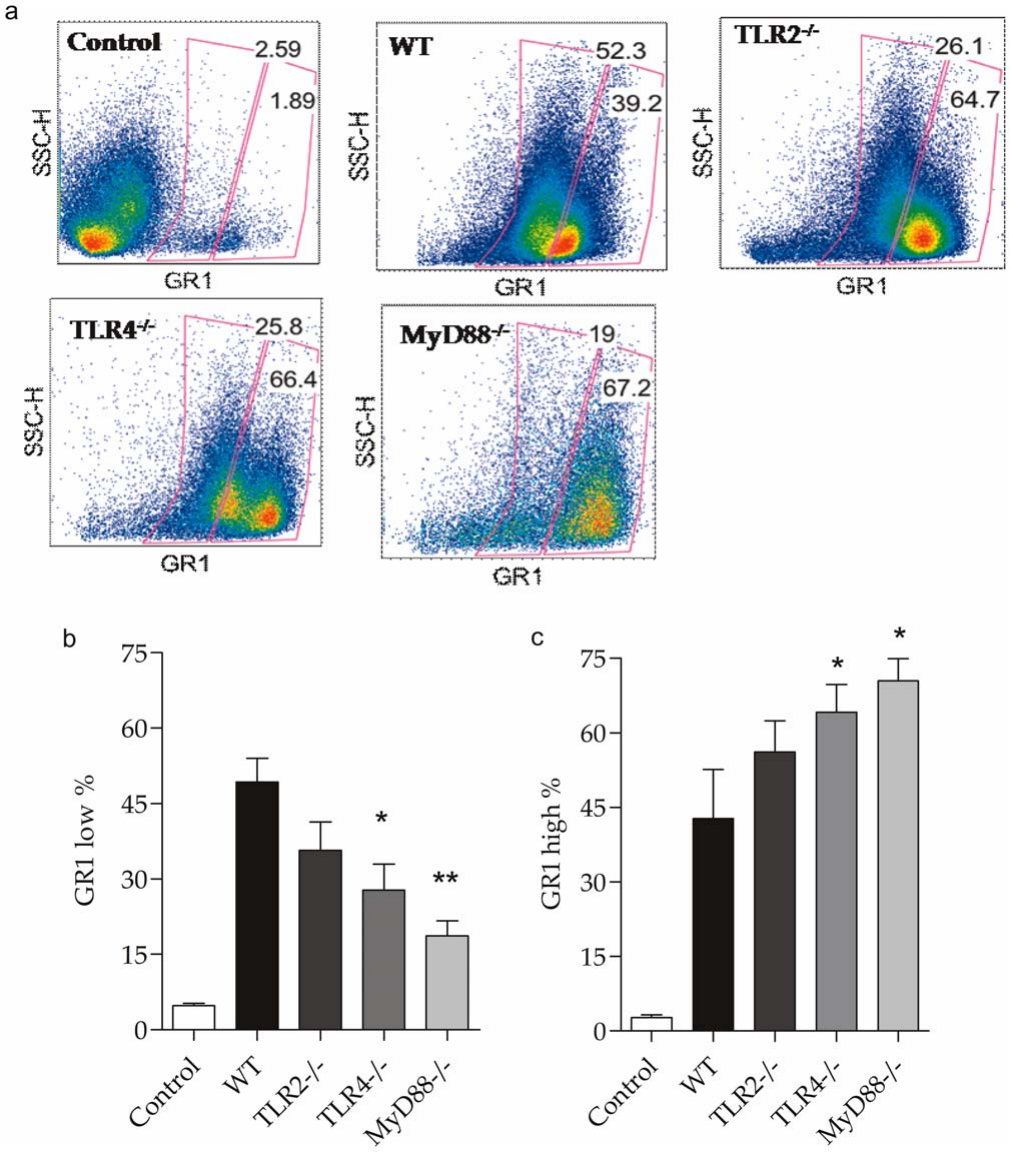
Migration GR1^+^ neutrophils into the peritoneal cavity after CLP. (a) Flow cytometry of the peritoneal cavity 24 hours after CLP in control, WT, TLR2^−/−^, TLR4^−/−^ and MyD88^−/−^ mice. (b, c) Frequency of GR1^low^ and GR1^high^ cells population in control, WT, TLR2^−/−^, TLR4^−/−^ and MyD88^−/−^ mice 24 hours after CLP. Data shown as mean ± standard deviation (SD).* p<0.05 vs. WT and ** p<0.01 vs. WT.

To investigate whether the absence of the receptors of innate immunity contributes to the local inflammatory response in sepsis through the migration of neutrophils to the peritoneal cavity, we analyzed the expression of the surface molecule GR1 in the peritoneal cavity after CLP. We observed significantly higher migration of neutrophils (GR1^+^^low^) to the peritoneal cavity after CLP in the WT mice compared with the TLR2^−/−^ , TLR4^−/−^ and MyD88^−/−^ knockout mice (**Figure 10 a and b**). The population with high expression of GR1^+^ (GR1^+high^) was significantly decreased in the peritoneal cavity of the WT mice compared with the other knockouts (**Figure 10a and c**). The population of GR1^+low^ cells was smaller in the peritoneal cavity after sepsis in the WT mice [12], but this population was also smaller in the knockout mice, which had a higher migration of GR1^+^^high^ cells. The absence of innate immune receptors resulted in decreased migration of the GR1^+low^ population and increased migration of the GR1^+high^ population to the peritoneal cavity, suggesting a role for these cells in the initial response to infection in the absence of TLRs.

### Effect of TLR2, TLR4 and MyD88 deficiency on neutrophil migration to the kidney after CLP

**Figure 11:**
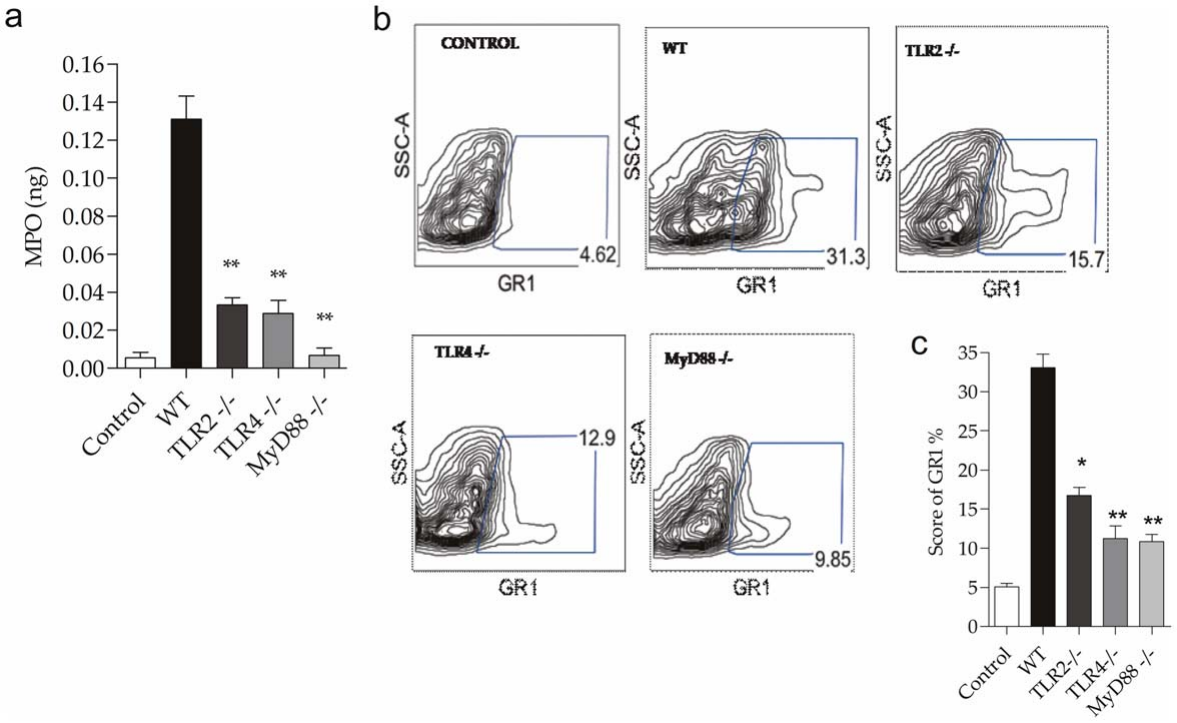
Neutrophil infiltration in renal tissue 24 hours after CLP. Gene expression of KC (a), IL-17 (b) , iNOS (c) in kidney 24 hours after CLP in WT, TLR2^−/−^ , TLR4^−/−^ and MyD88−/− mice. The mRNA was normalized to HPRT expression and compared to normal group. (d) Assay of myeloperoxidase activity in kidney of control, WT, TLR2^−/−^, TLR4^−/−^ and MyD88^−/−^ mice 24 hours after CLP. (e) GR1 expression in the kidney of mice 24 hours after CLP. (f) Results of a representative experiment with five animals/group. Data shown as mean ± standard deviation (SD).* p<0.05 vs WT and ** p<0.01 vs WT. ND: not detected.

To study whether the absence of TLRs and MyD88 leads to reduced neutrophil migration to the kidney after CLP, we first analyzed the expression of IL-17 and KC in the kidneys data shown in figure 4. We also evaluated the presence of neutrophils in the kidney by testing the myeloperoxidase activity. We observed that WT animals had a significant increase in myeloperoxidase activity compared with the control mice, and the MyD88^−/−^ cells had myeloperoxidase activity similar to that of normal controls (**Figure 11a**). We also observed a significant decrease in neutrophil migration to the kidneys of the TLR2^−/−^, TLR4^−/−^ and especially MyD88^−/−^ mice, as measured by GR1 by flow cytometry of renal tissue (**Figure 11b and c**).

### Neutrophil depletion in WT mice leads to amelioration of AKI after sepsis

**Figure 12:**
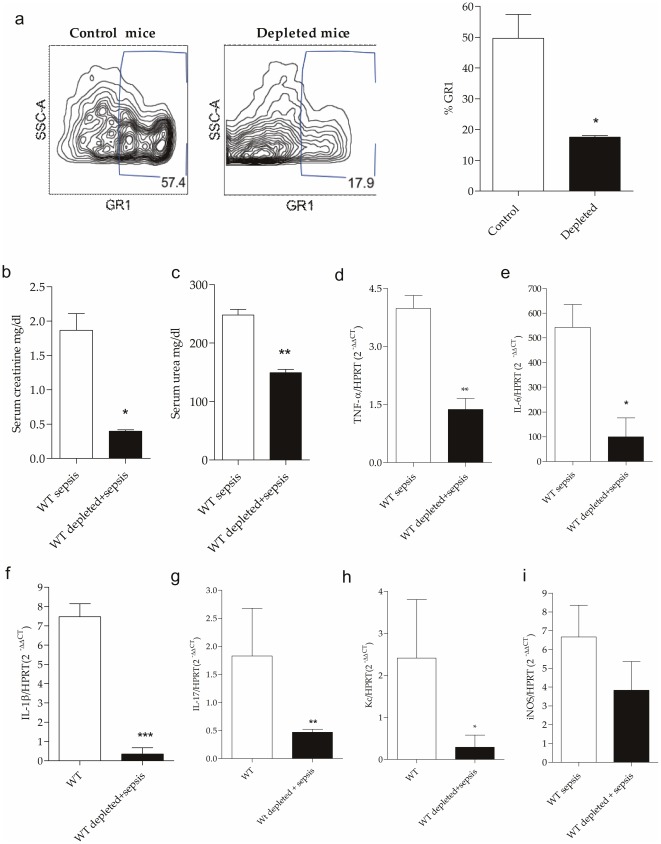
Partially neutrophils depletion in WT mice subjected to CLP. (a) Flow cytometry of spleen to determine the success of depletion in control mice without depletion in mice only depleted and depleted mice subjected to sepsis (*p<0.05 vs control). (b, c) Kidney function of WT mice and WT depleted mice subjected to CLP at time 24 hours, assessed by levels of serum creatinine and blood urea (*p<0.05 vs WT sepsis; **p<0.01 vs WT sepsis). (d, e, f, g, h, i) Gene expression of TNF-α, IL-6, IL1-β, IL-17, KC and iNOS in the kidney of WT mice and WT depleted mice 24 hours after CLP. The mRNA was normalized to HPRT expression and compared to normal group. Results of a representative experiment with three animals/group. Data shown as mean ± standard deviation (SD). *p<0.05 vs WT sepsis and **p<0.01 vs WT sepsis.

The decreased migration of neutrophils to the kidneys of the knockout mice after sepsis led us to hypothesize that neutrophils are also the main immune cells involved in the pathogenesis of AKI secondary to sepsis. Based on this hypothesis, we depleted neutrophils. We observed a significant decrease in spleen neutrophils in mice treated with RB6-8C5, demonstrating that depletion was achieved (**Figure 12a**). We found that the depleted mice had a significant improvement in renal function (**Figure 12b and c**). We also observed a significant decrease in the expression of TNF-α, IL-6, IL-1β, IL-17, KC and iNOS 24 hours after CLP (**Figure 12d and e**). These results suggested that an absence or even decrease in neutrophils confers protection against sepsis-induced AKI.

## Discussion

Our data suggest that hypotension during sepsis leads to tissue hypoxia, which could activate tubular and endothelial cells, culminating in the release of cytokines and chemokines. This release results in the increased vascular permeability that in turn can lead to the necrosis or apoptosis of tubular cells. Indeed, we observed tubular hypoxia as well as acute tubular necrosis and apoptosis in the WT mice. Hypoxia is considered a critical factor in organ dysfunction in sepsis [13]. Apart from hypotension, NF-κB also regulates the expression of HIF1-*α* [14], which increases in renal injury. The gene expression analysis of HIF1-α and qualitative analysis of hypoxia in the kidneys suggested that the TLRs and especially MyD88 are involved in renal injury. Some studies have shown that even without lowering blood pressure, the AKI induced by LPS or by other toxins results in the release of inflammatory mediators [15], [16]. Changes in vascular permeability are also important in the pathogenesis of sepsis-induced organ injury after significant production of cytokines, which lead to hemodynamic imbalance [17]. Renal vascular permeability increased after CLP in the WT mice, whereas in the TLR2^−/−^, TLR4^−/−^ and MyD88^−/−^ mice, vascular permeability was similar to control levels. Increased vascular permeability can cause a compression of peritubular capillaries [18], hemoconcentration and decreased microvascular flow.

We found that the MyD88-deficient mice were fully protected from damage caused by sepsis. The protection of renal function was not observed in the TLR2^−/−^ or TLR4^−/−^ mice when we analyzed the biochemical parameters, but there was a significant improvement in ATN in these mice compared with WT by renal histology, suggesting that TLR2 and TLR4 are required during polymicrobial sepsis. However, the absence of ATN, the significant decrease of the functional parameters and increased expression of MyD88 in the WT mice after CLP suggested that the mechanism of pathogen recognition in the kidney by both TLR2 and TLR4 occurs primarily through the adapter molecule MyD88. Recently, in addition to histological and serum markers, the molecule KIM-1 [19] was described as a marker of kidney damage. Corroborating our findings of ATN, we also observed a significant decrease of KIM-1 in the TLR2^−/−^ and TLR4^−/−^ and especially in the MyD88^−/−^ mice. Thus, we showed that the innate immunity receptors TLR2 and TLR4 and the adapter protein MyD88 are important in the development of AKI secondary to sepsis.

Wolfs and colleagues showed that TLR2 and TLR4 are constitutively expressed predominantly in the renal epithelial cells of distal and proximal tubules, the epithelium of Bowman's capsule and glomerular and endothelial cells, and the expression increases during the inflammatory process in the presence of IFN-γ and TNF-α [20]. In kidney tissue after sepsis, TLR2 and TLR4 mRNA is highly expressed in the WT mice, which also have a six-fold increased expression of MyD88. In the TLR2^−/−^ mice, we observed an overexpression of TLR4 compared with WT. It has been suggested that TLR2 is also responsible for LPS recognition [21], and in its absence, TLR4 is overexpressed. This may account for the lack of AKI in the MyD88^−/−^ mice.

TLRs may also be activated during sepsis by alarmins. Previous studies showed increased levels of HMGB1 and HSP70 in conditions of cell damage and inflammation [4], [9]. We showed that these ligands are highly expressed in the WT mice and have decreased expression in the knockouts mice after sepsis. These ligands can cause robust production of cytokines, and consequently the development of AKI in septic mice.

IL-1β, TNF-α, and IL-6 were decreased in the kidneys of the TLR2^−/−^, TLR4^−/−^ and especially in MyD88^−/−^ mice as a consequence of decreased NF-κB p65 translocation to the nucleus. Previous studies with MyD88^−/−^ mice showed improved renal function of these animals after sepsis and also decreased serum levels of TNF compared with controls [7]. Furthermore, studies have shown that levels of TNF-α, IL-1β, and IL-6 are markedly increased in patients with established sepsis [22]. In the kidney, endotoxin causes mesangial cells to release TNF-α [23], [24]. LPS treatment in mice deficient in the TNF receptor caused lower renal tubular apoptosis and infiltration of neutrophils compared with WT mice [25], [26].

Recently, the role of apoptosis in the pathogenesis of sepsis has also been explored. Administration of the caspase inhibitors or overexpression of the anti-apoptotic protein Bcl-2 significantly improves the survival of CLP-induced septic mice [27], [28], [29]. Our study showed that septic MyD88^−/−^ mice have high expression of the anti-apoptotic molecule BCL-2 in the kidney and absence of apoptosis, indicating a possible mechanism that inhibits apoptosis in these animals.

Sepsis results in decreased migration of neutrophils to the infection site, hindering the removal of pathogens and initiating a robust inflammatory response characterized by an inadequate sequestration of neutrophils to organs [30]. We believe that in our model, the renal dysfunction was due to an exaggerated response to infectious stimuli and the activation and recruitment of neutrophils to the kidney. Andonegui et al. demonstrated that the absence of CD14 and TLR4 prevented neutrophil sequestration to the lungs after treatment with LPS [31], and Goseman et al. also described the impairment of neutrophil migration to the lungs of mice deficient in TLR4 [32]. Alves-Filho et al. showed that deficiency in the migration of neutrophils to the infection after CLP is associated with increased mortality [33] in TLR4 deficient mice. Alves-Filho et al. also showed that TLR2 has a detrimental role in polymicrobial sepsis and suggested that the inhibition of TLR2 signaling may improve survival in sepsis [34].

We found increased TNF-α secretion by macrophages in the peritoneal cavity in the WT mice compared with the knockouts mice. The significant synthesis of TNF-α is responsible for the severe sepsis and recruitment of neutrophils to the infectious focus. Surprisingly, we found a significant decrease in the infiltration of the GR1^+low^ population, described as resident macrophages [35], in the peritoneal cavity of knockout mice compared with WT mice. Furthermore, we found a significant decrease in the GR1^+high^ population, which some authors describe as granulocytes, in the peritoneal cavity of the WT mice. Miyazaki and colleagues showed that 60 min post injection of LPS, the proportion of GR1^+high^ decreased and this process was depended on TNF-α. Therefore, the decrease of GR1^+high^ coincided with the increase in cell accumulation in mesenteric lymph nodes [36]. This may be related to the activation of the inflammatory response by other pathways of pathogen recognition and the failure of migration of neutrophils to the peritoneal cavity in the WT mice after sepsis. This would also explain the migration of neutrophils in the knockout mice.

In the kidneys of knockout mice with sepsis, especially the MyD88 knockout mice, we found decreased expression of IL17, KC, iNOS, and MPO activity. Furthermore, the expression of GR1 decreased. We concluded that the migration of neutrophils to the kidney after sepsis is dependent on the activation of TLR and MyD88 and mostly decreases the release of pro-inflammatory cytokines. In septic mice depleted of neutrophils, we observed a significant improvement in renal function and decreased expression of TNF-α, IL-6, IL-1β, IL-17, KC and iNOS in the kidneys. Experimental models using antagonists and knockout mice that decrease the activity of neutrophils have shown improved organ function [37], [38], [39].

The TLRs are important for tissue protection against bacteria, but in a persistent infectious process, they can be harmful. Persistent inflammation can lead to kidney damage and production of alarmins, which may exacerbate the TLR activation of the inflammatory response and thus cause harmful positive feedback.

## Supporting Information

Figure S1 **mRNA expression of TLR2 (a), TLR4 (b), MyD88 (c) in the kidney of WT, TLR2^−/−^, TLR4^−/−^ and MyD88^−/−^ mice 24 hours after CLP.** The mRNA was normalized to HPRT expression. Results of a representative experiment with 5 animals per group. Data shown as mean ± standard deviation (SD), ND: Not Detected, *** p<0. 0001 vs WT; ** p<0.01 vs WT.
(TIF)

Data S1 **RT-PCR was performed using Taqman primers (Applied Biosystems, USA).**
(TIF)

Data S2 **RT-PCR was performed using Syber primers (Applied Biosystems, USA).**
(TIF)
