# Supplementary figures and images for: TLR2, TLR4 and the MYD88 Signaling Pathway Are Crucial for Neutrophil Migration in Acute Kidney Injury Induced by Sepsis
Source: PLoS One. 2012 May 24;7(5):e37584. doi: 10.1371/journal.pone.0037584 (PMC3360043; doi:10.1371/journal.pone.0037584)

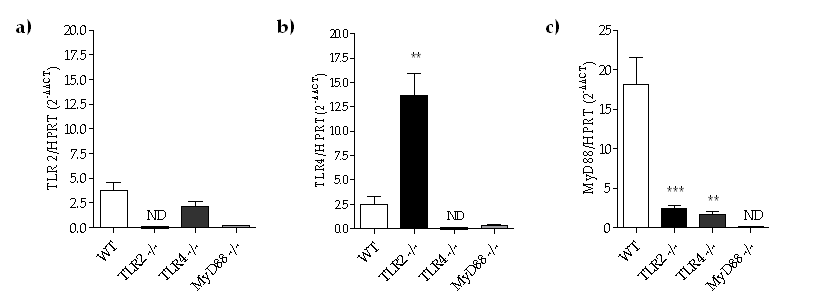

Supplement: Figure S1 — mRNA expression of TLR2 (a), TLR4 (b), MyD88 (c) in the kidney of WT, TLR2−/−, TLR4−/− and MyD88−/− mice 24 hours after CLP. The mRNA was normalized to HPRT expression. Results of a representative experiment with 5 animals per group. Data shown as mean ± standard deviation (SD), ND: Not Detected, *** p<0. 0001 vs WT; ** p<0.01 vs WT. (TIF) [file pone.0037584.s001.tif]

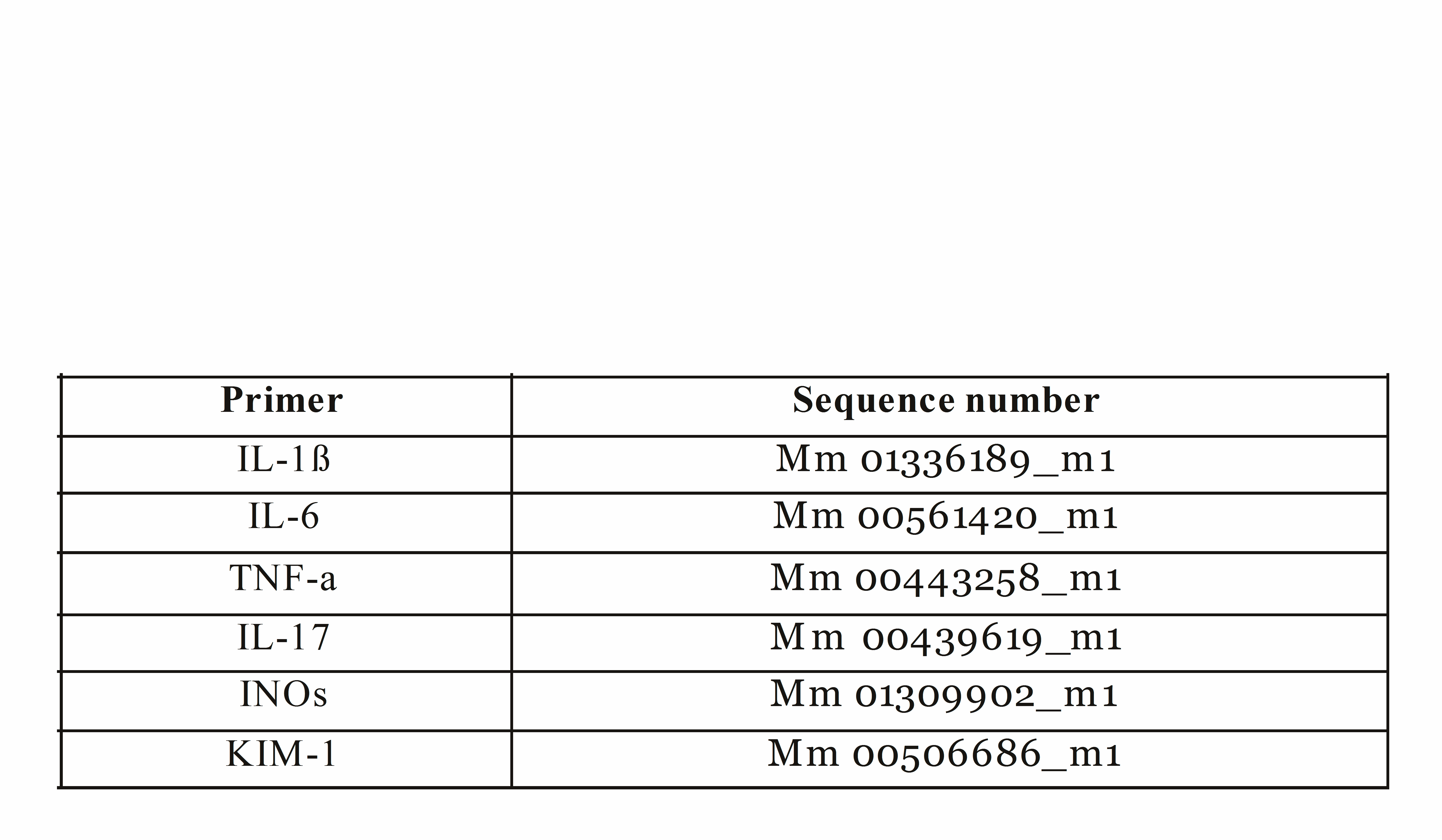

Supplement: Data S1 — RT-PCR was performed using Taqman primers (Applied Biosystems, USA). (TIF) [file pone.0037584.s002.tif]

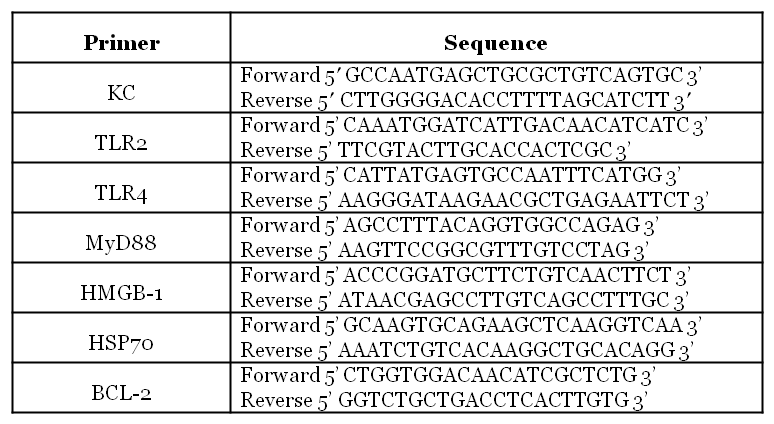

Supplement: Data S2 — RT-PCR was performed using Syber primers (Applied Biosystems, USA). (TIF) [file pone.0037584.s003.tif]
